# Morphological Examination and Phylogenetic Analyses of *Phycopeltis* spp. (Trentepohliales, Ulvophyceae) from Tropical China

**DOI:** 10.1371/journal.pone.0114936

**Published:** 2015-02-02

**Authors:** Huan Zhu, Zhijuan Zhao, Shuang Xia, Zhengyu Hu, Guoxiang Liu

**Affiliations:** 1 Key Laboratory of Algal Biology, Institute of Hydrobiology, Chinese Academy of Sciences, Wuhan, People’s Republic of China; 2 University of Chinese Academy of Sciences, Beijing, People’s Republic of China; 3 College of Life Sciences, South-central University for Nationalities, Wuhan, People’s Republic of China; Louisiana State University, UNITED STATES

## Abstract

During an investigation of Trentepohliales (Ulvophyceae) from tropical areas in China, four species of the genus *Phycopeltis* were identified: *Phycopeltis aurea*, *P. epiphyton*, *P. flabellata* and *P. prostrata*. The morphological characteristics of both young and adult thalli were observed and compared. Three species (*P. flabellata*, *P. aurea* and *P. epiphyton*) shared a symmetrical development with dichotomously branching vegetative cells during early stages; conversely, P. prostrata had dishevelled filaments with no dichotomously branching filaments and no symmetrical development. The adult thalli of the former three species shared common morphological characteristics, such as equally dichotomous filaments, absence of erect hair and gametangia formed in prostate vegetative filaments. Phylogenetic analyses based on SSU and ITS rDNA sequences showed that the three morphologically similar species were in a clade that was sister to a clade containing *T. umbrina* and *T. abietina*, thus confirming morphological monophyly. Conversely, *Phycopeltis prostrata*, a species with erect filaments, sessile gametangia on the basal erect hair, larger length/width ratio of vegetative cells and very loosely coalescent prostrate filaments, branched separately from the core *Phycopeltis* group and the *T. umbrina* and *T. abietina* clade. Based on morphological and molecular evidence, the genus *Phycopeltis* was paraphyletic. Furthermore, the traditional taxonomic criteria for *Phycopeltis* must be reassessed based on phylogeny using more species. A new circumscription of the *Phycopeltis* and the erection of new genera are recommended.

## Introduction

The genus *Phycopeltis* was established by Millardet (1870) with *P. epiphyton* Millardet as its type species [1]. *Phycopeltis* often constitutes an important part of epiphyllous growth in tropical and subtropical ecosystems. This genus grows not only superficially on leaves, twigs, fruits and stems of higher plants but also on non-living substrata in areas of high humidity [2]. Species in *Phycopeltis* are open-branched and show filamentous to pseudoparenchymatous growth. Members of *Phycopeltis* have a life history that probably consists of an isomorphic alternation of diploid sporophytes and haploid gametophytes [2, 3, 4, 5, 6, 7]. Thompson and Wujek (1997) emphasised the taxonomic value of several characteristics that were previously ignored: the arrangement of gametangia (scattered or arranged in concentric rings), relative distribution of zoosporangia and gametangia (occurring simultaneously on the same thallus or on separate thalli) and production of glandular papilla on the dorsal surface of cells [2, 7]. A feature that aids in distinguishing *Phycopeltis* from other foliicolous genera (*Trentepohlia*, *Printzina*, *Cephaleuros* and *Stomatochroon*) is the terminal papilla-pore on the sporangium, which is the opposite end of the attachment [2, 8]. In other genera, the papilla-pore is located at the base and adjacent to the area of attachment [2, 8]. Henrik Printz described 12 species of this genus [3]. The monograph of *Phycopeltis* prepared by Thompson and Wujek (1997) described 11 new species, a new combination [*P. umbrina* (Kützing) R.H. Thompson & D.E. Wujek] and 11 new varieties. The authors also treated *P. maritima* Karsten and *P. microcystis* Schmidle as synonyms for *P. epiphyton* [2]. Neustupa (2005) also described two new species from Southeast Asia [9]. Therefore, *Phycopeltis* Millardet currently includes 25 species (Table 1).

**Table 1:** Morphological data of 25 *Phycopeltis* species from previous studies.

**species**	**length/width (G/S)**	**size of vegetative cell (G/S)**	**ramulli shape**	**gametangia**	**sporangia**	**erect sterile hairs**
*Phycopeltis amboinensis*	_/3.0–4.5	_/6.0–9.0×22.0–32.0	open-branching and flabelllate	terminal or intercalary	13.4–16.0×22.4–25.7, intercalary or marginal	with
*Phycopeltis arundinacea*	_/3.5–4.5	_/7.0–12.0×28.0–38.0	circular disk	intercalary or centripetal	15–22.3×19.6–26.4, intercalary	without
*Phycopeltis aurea*	1.6–2.8/19.–2.1	2.5–4.2×6.4–8.5/3.9–4.6×8.2–9.6	circular disk	terminal or intercalary	12–15×13.8–16.6, intercalary	without
*Phycopeltis costaricensis*	1.6–3.2	4.6–8.0×11.4–14.8	open-branching, with three or more central filaments	intercalary/subterminal or centripetal	12.8–15.6×16–18.6, intercalary	without
*Phycopeltis dorsopapillosa*	_/1.75–2.8	_/8.0–15.0×22.4–26.4	circular disk	intercalary	16.6–27.4×18.6–29.4, intercalary	without
*Phycopeltis epiphyton*	3.0/3.0	4.2–6.3×8.4–18.0/4.6–7.7×15.0–26.0	circular disk	intercalary, 8.0–14.6×16.3–20.0	14.2–16.6×18.0–24.6, terminal or intercalary	without
*Phycopeltis expansa*		8.0–12.0×13.0–18.0	disk with lobed margins	intercalary, 13.0–22.0×22.0–26.0	14.0–26.0×17.0–26.0, terminal	without
*Phycopeltis flabelligera*			circular disk			without
*Phycopeltis falbellta*	2.0/2.6	5.7–7.4×11.6–12.9/5.6–8.8× 16.8–22.0	laterally appressed and flabellate	terminal	12.0–15.3×14.0–17.6, submarginal	without
*Phycopeltis irregularis*	_/1–2.0	4.6–8.4×13.0–16.2	branched-filamentous, irregularly widespreading	intercalary	8.0–9.4×11.6–14.0, intercalary	without
*Phycopeltis javanica*	2.0–2.9/1.7–3.1	4.7–8.9×8.7–17.5/7–13.2×9.4–25.5	circular disk with crenate or irregular lobed sinsues	intercalary or centripetal, 6.2–14.0×11.5–25.0	intercalary, 12.5–20.8×14.0–21.5	without
*Phycopeltis juarensis*	1.5–2.7/2–4.4	3.7–7.6×8.0–12.5/3.5–8.0×9–22.5	circular disk	intercalary or centripetal, 4.8–13.0×8.5–25.5	terminal, 10.0–17.5×10.5–18.8	without
*Phycopeltis kosteriana*	2.3–3.2/_	2.2–3.8×7.0–12.0/_	irregular disk composed of loose prostrate filaments	intercalary		without
*Phycopeltis minuta*		3.8–7.7×9.6–19.2	filaments open and wide-spreading, irregular branches	intercalary or terminal, 3–5×6–7.5	intercalary, 9.4–11.2×10–12.4	without
*Phycopeltis nigra*	2.4–3.5	4–11×15–19	open-branching and flabelllate		intercalary or submarginal, 11.3–14.8×14.4–16.0	without
*Phycopeltis novae-zealandiae*	1.6–3.9/1.8–2.8	4.5–7.5×12.5–17.6/5.5–8.5×16–22	circular disk	interclary and concentric	intercalary, 10.6–15.8×12.4–18.6	without
*Phycopeltis parva*		2.5–8.4×10.3–20.0	without definite form, irregular branches	intercalary, 8.7–16.2 in diameter	apical or terminal, 15.0–21.0 ×18.7–27.5	without
*Phycopeltis pilosa*	3.0–4.6/_	3.9–7.7×12.0–24.0	circular disk	terminal		without
*Phycopeltis prostrata*	2.1–5.2	2.4–5.0 × 6–13.5	circular disk with loosely prostrate filaments	on the basal cell of erect filaments, 4.6–7.1× 9.4–10.8	intercalary, 5.2–7.8 in diameter	with
*Phycopeltis pseudotreubii*	1.9–2.9/_	4.6–6.4×12.0–15.8/_	circular disk with crenate or irregular lobed sinsues	mostly intercalary, also terminal	intercalary, 10.6–11.4×13.0–14.8	without
*Phycopeltis terminopapillosa*	2.1–2.8/_	5.8–7.2 ×13.4–17.2/_	disk with flabellate ramulli	terminal	intercalary, 10.8–12.4 ×13.4–16.4	without
*Phycopeltis theaensis*	1.5–2.8/1.5–2.4	4.6–8.7 ×7.5–17.5/6.2–11.5 ×10.0–23.6	circular disk	intercalary, 8.3–16.4 ×13.7–25.0	itercalary, 15.0–18.3 ×16.2–22.7	without
*Phycopeltis treubii*	2.2–3.2	7–14×12–35	disk with flabellate ramulli	terminal	intercalary, 13.8–15.8×14.4–18.2	without
*Phycopeltis treubioides*	1.66–2.9	5.2–10.3×14.9–18.6	disk with flabellate ramulli	terminal	intercalary or terminal, 22–23.7×25.5–34.7	without
*Phycopeltis vaga*	1.75–3.5	4–11×15–19	open-branching and flabelllate	terminal or medial	intercalary, 11.3–14.8×14.4–16.0	without

G = gametophyte; S = sporophyte.

Numerous studies have focused on the systematic and phylogenetic position of the order Trentepohliales. Raven (1987) classified the Trentepohliales in the class Pleurastrophyceae, whereas Sluiman (1989) suggested that the Trentepohliales should be regarded as an order in the class Ulvophyceae [10, 11]. The monophyly of the Trentepohliales is expected because some features such as the sporangium-associated apparatus and the flagellar apparatus are unique to this order [8]. Trentepohliales is recovered as the sister lineage of the Bryopsidales, Cladophorales and Dasycladales, together referred to as the Trentepohliales, Bryopsidales, Cladophorales and Dasycladales (TBCD) clade [12]. However, previous phylogenetic studies of Trentepohliales have focused on genera from which the species were easily cultured, including *Trentepohlia*, *Printzina* and *Cephaleuros*.

The phylogeny based on the molecular data of the genus *Phycopeltis* is scant because attempts to establish cultures of *Phycopeltis* have failed. However, studies of Nelsen et al. [13] and Hametner et al. [14, 15] were dealing with lichenized Trentepohlialean algae which were not cultured. Undoubtedly, these environmental sequences may include some *Phycopeltis* sequences. Overall, several questions concerning this genus remain unresolved. These questions include whether *Phycopeltis* is a monophyletic or paraphyletic group and its relationship with other genera, and which morphological characteristics can be useful in delimitation for this genus. In the present study, we address these questions by observation of vegetative and developmental morphology and phylogenetic analyses based on nuclear-encoded SSU and ITS rDNA sequences of several species sampled from tropical and subtropical areas of China.

## Material and Methods

### Sampling

*Phycopeltis* samples used in this study were obtained from the Xishuangbanna Tropical Botanical Garden, Chinese Academy of Sciences in Yunnan, as well as Nanling National Forest Park in Guangdong on 23 June and 28 August 2012, respectively. For our collection, no specific permissions were required in these two gardens. Leaves and some plastic tags on which *Phycopeltis* were epiphytic were removed by a scissors (S1 Fig). The samples collected in the field were preserved in plastic pockets back to the laboratory and dried as herbarium specimens. Some samples were preserved in the field using silica gel. Those five voucher specimens were deposited in the Freshwater Algal Herbarium (IHB), Institute of Hydrobiology, Chinese Academy of Sciences, Wuhan, China.

### Morphological observation

The developmental stages of *Phycopeltis* species were determined by examining various discrete stages found in the field material. Free-hand sections were made under a stereoscope (Zeiss model KL1500 LCD; Carl Zeiss, Göttingen, Germany) to obtain pure material. Substratum slices (mainly leaves) were then soaked overnight in NaOH (0.6 M). The same volume of HCl as NaOH (0.6 M) was used to neutralise the substratum. The thalli of the *Phycopeltis* were easily stripped from the leaves and repeatedly rinsed with sterile water. The morphological traits and microstructure were observed under a microscope (Leica model DM5000B; Leica Microsystems GmbH, Wetzlar, Germany). About 60 cells per species were measured in our observation. The specimens were stained with 0.1% Fluorescent Brightener 28 (Sigma-Aldrich, Poole, UK) to observe young thalli by epifluorescence microscopy (Leica model DM5000B). The method was described in detail by Fritz and Triemer [16] and Liu et al. [17]. Micrographs were taken with a Leica DFC320 digital camera. Fixed specimens from field samples were cut into small squares under a stereoscope for scanning electron microscopy. These samples were then dehydrated in a tert-butanol series (50, 70, 90 and 100%) for 15 min each and then critical point-dried. The dried plants were mounted on stubs, coated with gold for 30 s and observed under a scanning electron microscope (Hitachi model S-4800; Hitachi Ltd., Tokyo, Japan).

### DNA extraction and PCR amplification

The unialgal mass (about 15–20 thalli) was added to 1 mL of 0.5-mm glass beads and 1 mL of phosphate buffer solution (PBS, pH 7.0). The algal cells were lysed by bead beating at 4800 rpm for 2 min in a mini-beadbeater (Model 3110BX, Biospec Products, Bartlesville, Oklahoma USA). For total genomic DNA extraction, the lysates were treated as described in the AxyPrep Multisource Genomic DNA Miniprep Kit (Axygen Biotechnology, Hangzhou, China) handbook.

The universal primers designed by Medlin et al.[18] were used to amplify the partial nuclear-encoded SSU rDNA sequences. The SSU rDNA sequence amplification profile consisted of a preliminary 3 min denaturing at 95°C, 30 cycles of 50 s denaturing at 94°C, 50 s annealing at 56°C, 90 s extension at 72°C and a final extension of 7 min at 72°C. The excised PCR products were cloned into a pMD18-T vector (Takara Bio Inc., Otsu, Shiga, Japan). One to five clones of each PCR products were selected for sequencing respectively. Universal primers M13F (5ʹCGCCAGGGTTTTCCCAGTCACGAC3ʹ) and M13R (5ʹAGCGGATAACAATTTCACACAGGA3ʹ) were used for sequencing [19]. Polymerase chain reaction and sequencing of the ITS1-5.8S-ITS2 region were carried out as Hayakawa et al.[20]. The clones of SSU rDNA sequence and PCR products of ITS rDNA sequence were sent to WuHan Tsingke BioTech Co. Ltd. (WuHan, China) for sequencing. A blast research was made to rule out possible contaminants. And six SSU and seven ITS rDNA sequences of *Phycopeltis* were aligned for further analyses.

### Phylogenetic analyses

Trentrepohlialean algae were selected from GenBank (http://www.ncbi.nlm.nih.gov/) for nuclear SSU and ITS rDNA sequences analyses. The sequences were initially aligned by ClustalX (v. 1.83) [21], and refined manually with Seaview (v. 4.32) [22]. The final alignment of SSU and ITS rDNA sequences comprised a matrix of 41 sequences and 33 sequences respectively (Supplementary materials). Mutational saturation was evaluated in the variable positions of the alignments by plotting pairwise distances against model-corrected distances for Tamura and Nei (1993) and Kimura (1980) models and estimated in MEGA (v.5.0) [23]. And the result showed that neither transitions nor transversions have reached saturation. The empirical base frequencies of the nuclear SSU and ITS rDNA sequences were determined by PAUP 4.0*b10 [24] to test for homogeneity of base frequencies across the taxa.

Phylogenetic trees were constructed using maximum parsimony (MP) with PAUP 4.0*b10 [24], maximum likelihood (ML) with RAxML8.0 (http://www.exelixis-lab.org/web/software/raxml/hands_on.html) [25], and Bayesian Inference (BI) with MrBayes3.1.2 [26]. ModelTest 3.7 [27] was used to select the evolutionary best-fit model according to hierarchical likelihood ratio tests and Akaike information criterion. The best-fit models of both SSU rDNA and ITS rDNA data was GTR+I+G. Bootstrap analyses with 1000 replicates of the dataset for ML and MP was performed to estimate statistical reliability. Bayesian analyses of both SSU rDNA and ITS rDNA sequence was performed with 1.0× 10^7^ generations of Markov chain Monte Carlo iterations and trees were sampled every 1 × 10^4^ generations. It was assumed that the stationary distribution was reached when average standard deviation of split frequencies between two runs was lower than 0.01. The first 25% of the calculated trees were discarded as burn-in, and the remaining samples were used to construct a Bayesian consensus tree and to infer posterior probabilities. The bootstrap values and posterior probabilities were presented on the nodes. The resulting phylogenetic trees were showed and edited using Figtree1.4.2 (http://tree.bio.ed.ac.uk/software/figtree/). Only maximum likelihood trees were showed.

## Results

### Morphology and Taxonomy (Figs. 1–5)

**Fig 1:**
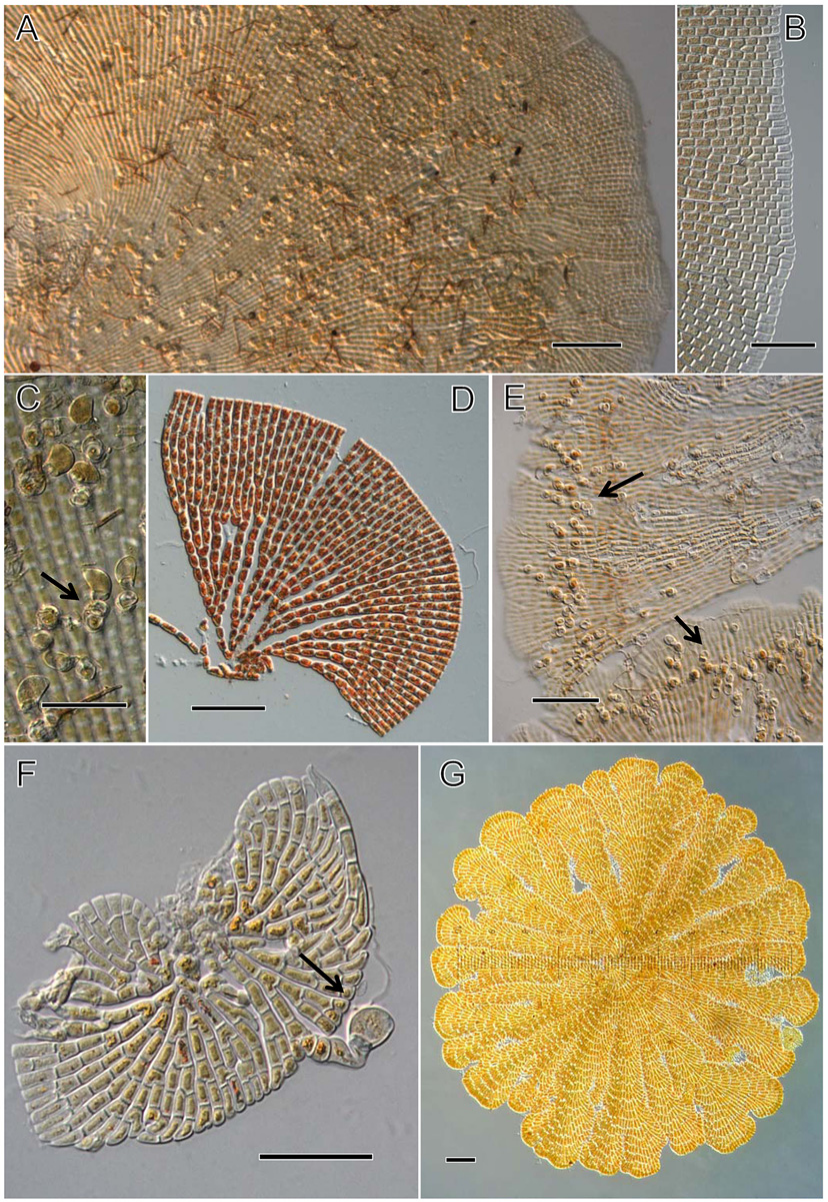
Microscopic features of *Phycopeltis aurea*, *Phycopeltis epiphyton* and *Phycopeltis flabellata*. Fig. 1A–1C. Microscopic features of Phycopeltis aurea. Bars = 50 μm. Fig. 1A. Surface view of P. aurea. Fig. 1B. Discoidal thallus with an even, crenate margin. Fig. 1C. Intercalary sporangiate-laterals. Fig. 1D–1F. Microscopic features of Phycopeltis epiphyton. Bars = 50 μm. Fig. 1D. Surface view of the filaments of P. epiphyton. Fig. 1E. Sporangiate-lateral (arrows) lying on the submarginal positions of discoid thalli. Fig. 1F. Sporangiate-lateral developing from an apical cell on a compressed thallus (arrow). Fig. 1G. P. flabellata consisting of compressed ramuli.

**Fig 2:**
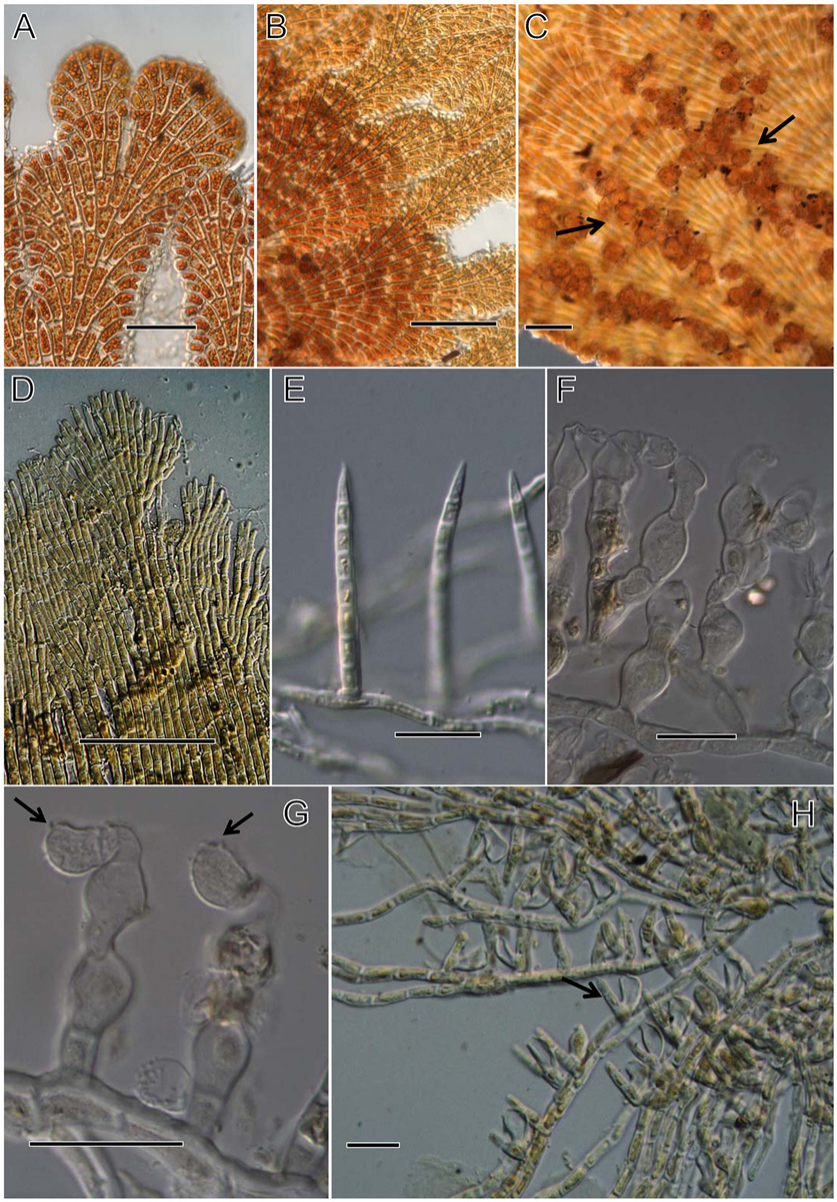
Microscopic features of *Phycopeltis flabellata* and *Phycopeltis prostrata*. Fig. 2A–2C. Microscopic features of *Phycopeltis flabellata*. Bars = 50 μm. Fig. 2A. The fan-like ramuli of *P. flabellata*. Fig. 2B. New growth starting from the margins of the older ramuli. Fig. 2C. Sporangia of *P. flabellata* on the margins of fan-like ramuli. Fig. 2D–2H. Microscopic features of *Phycopeltis prostrata*. Bars for Fig. 2D, 50 μm; others, 20 μm. Fig. 2D. Disjunct margin with dichotomous filaments of *T. prostrata*. Fig. 2E. Attenuate erect hair composed of eight cells. Fig. 2F–2G. Few-celled (usually two) stalked sporangia with a terminal papilla (arrows) developing from the prostrate filaments. Fig. 2H. Sessile gametangia (arrows) on the base of erect filaments.

**Fig 3:**
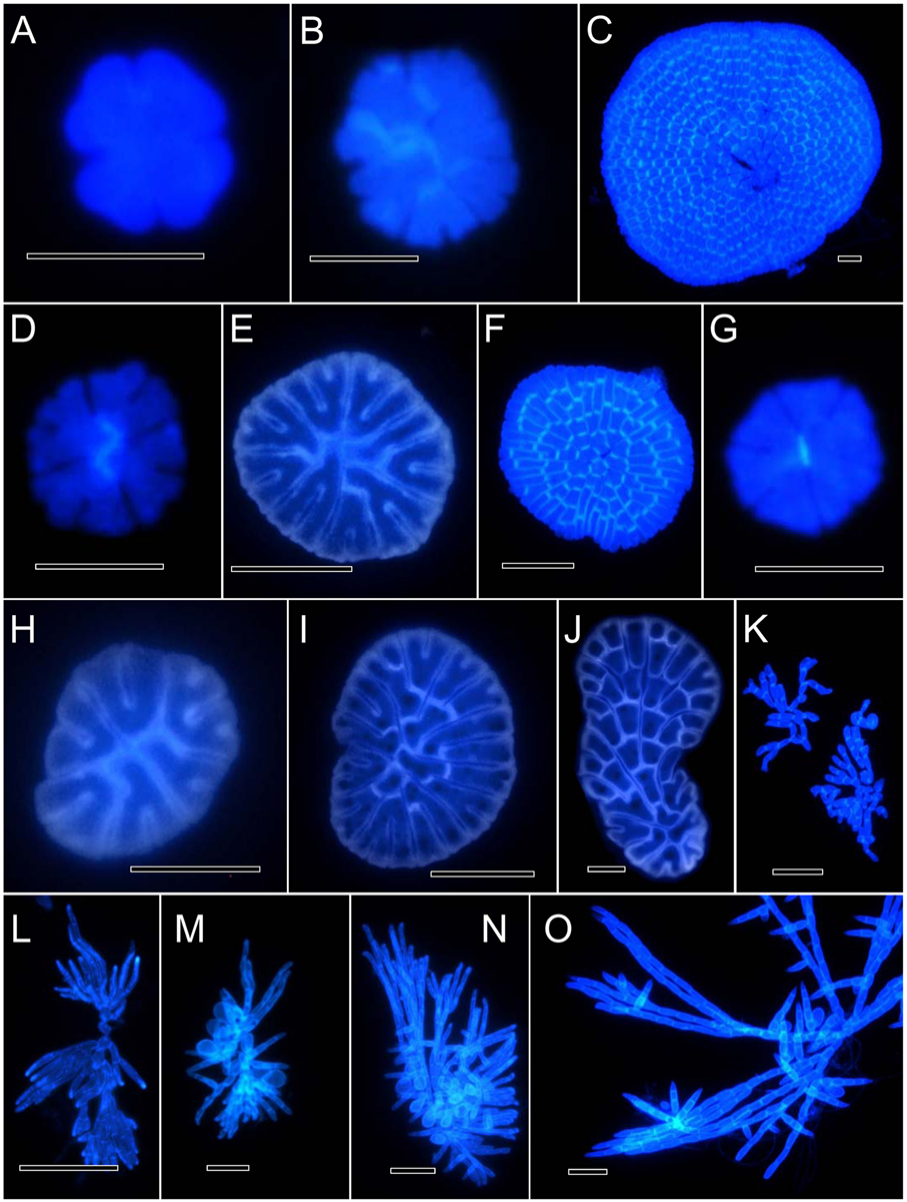
Development of the four species: *P. aurea*, *P. epiphyton P. flabellata*, and *P. prostrata*. Fig. 3A–3C. Development of *P. aurea*. Bars = 10 μm. Fig. 3D–3F. Development of *P. epiphyton*. Bars = 10 μm. Fig. 3G–3J. Growth of *P. flabellata*. Bars for Fig. 3G, 5 μm, for others, 10 μm. Fig. 3K–3O. Development of *P. prostrata*. Bars = 20 μm.

**Fig 4:**
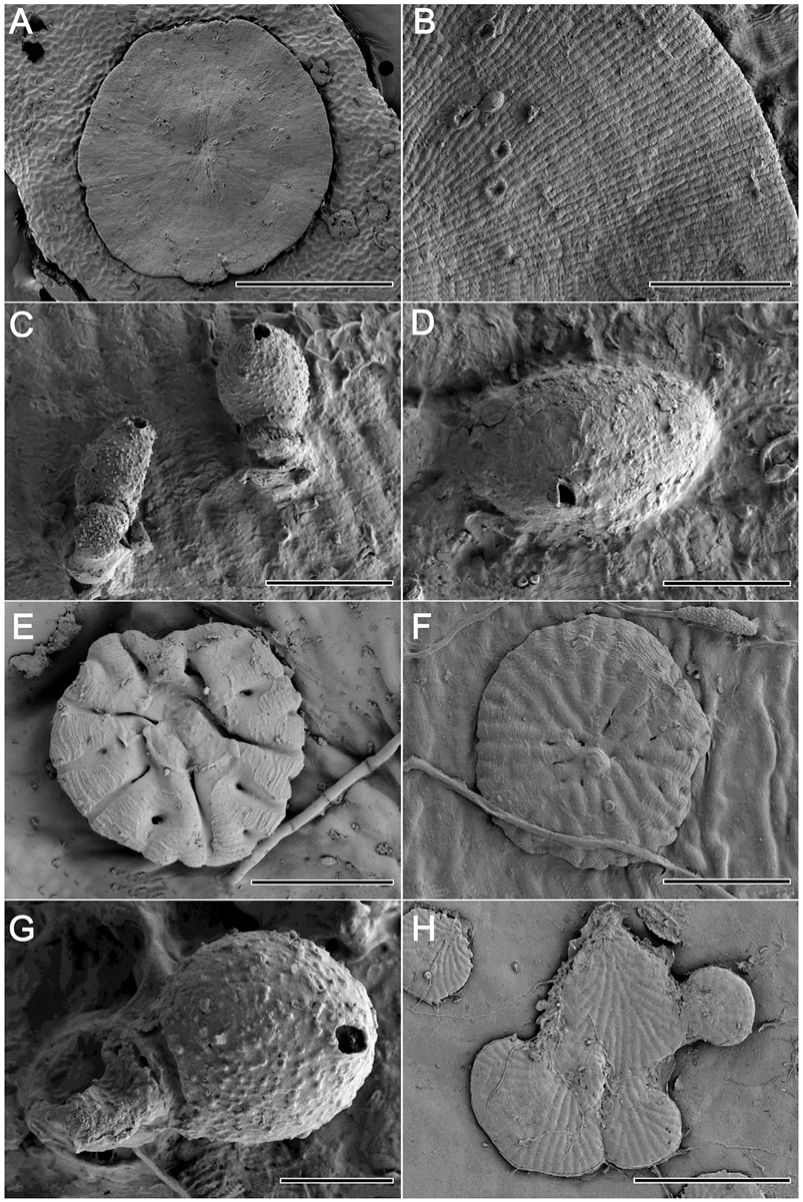
SEM images of *Phycopeltis aurea*, *P. epiphyton* and *P. flabellata*. Fig. 4A. Gametophyte thallus of *P. aurea*. Bar = 500 μm. Fig. 4B. Marginal thallus of *P. aurea*. Bar = 100 μm. Fig. 4C. Sporangia of *P. aurea*. Bar = 20 μm. Fig. 4D. Gametangium of *P. aurea*. Bar = 10 μm. Fig. 4E. Young thallus of *P. epiphyton*. Bar = 10 μm. Fig. 4F. Thallus of *P. epiphyton*. Bar = 30 μm. Fig. 4G. Sporangium of *P. epiphyton*. Bar = 10 μm. Fig. 4H. Young thalli of *P. flabellata*. Bar = 100 μm.

**Fig 5:**
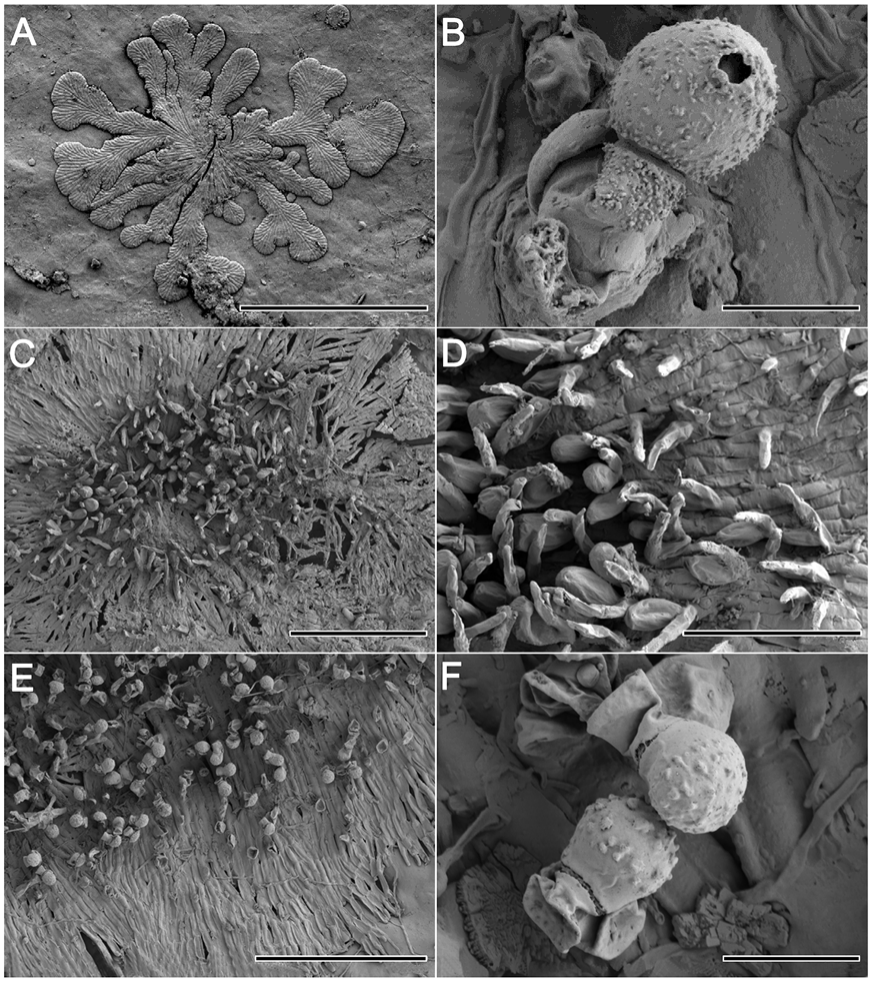
SEM images of *Phycopeltis flabellata* and *P. prostrata*. Fig. 5A. Adult thallus of *P. flabellata*. Bar = 500 μm. Fig. 5B. Sporangium of *P. flabellata*. Bar = 10 μm. Fig. 5C. Thallus of *P. prostrata* with erect hair. Bar = 100 μm. Fig. 5D. Sessile gametangia of *P. prostrata*. Bar = 40 μm. Fig. 5E. Partial thallus of sporophyte with sporangia. Bar = 100 μm. Fig. 5F. Sporangia of *P. prostrata*. Bar = 10 μm.

Four species were identified from our collections: *Phycopeltis aurea* Karten, *Phycopeltis epiphyton* Millardet, *Phycopeltis flabellata* R.H. Thompson & D.E. Wujek and *Phycopeltis prostrata* (De Wildeman) Schmidle. Furthermore, a comparison of *Phycopeltis* morphological data based on our observation and previous studies was made in Table 1, and a comparation of key characters of species used in present phylogenetic analyses was also made in table 2 [1, 2, 3, 7, 9, 28, 29, 30, 31, 32, 33, 34, 35, 36, 37, 38].

**Table 2:** Key characters of species in present phylogram used to distinguish from other Trentepohliacean genera.

**Species**	**heterotrichous thallus**	**rhizoids**	**dichoutomous filaments**	**papilla-pore**	**sporangium**	**gametangium**
*Cephaleuros virescens*	yes	with	yes	basal	in cluster	lateral
*Cephaleuros parasiticus*	yes	with	yes	basal	in cluster	lateral
*Phycopeltis aurea*	no	without	yes	distal	solitary	intercalary or terminal
*Phycopeltis epiphyton*	no	without	yes	distal	solitary	intercalary or terminal
*Phycopeltis flabellata*	no	without	yes	distal	solitary	intercalary or terminal
*Phycopeltis prostrata*	yes	without	yes	distal	solitary	intercalary or terminal
*Physolinum monile*	no	without	no	-	-	-
*Printzina bosseae*	no	without	no	distal	solitary	-
*Printzina lagenifera*	no	without	no	basal	in cluster or solitary	sessile or lateral
*Tretepohlia abietina*	no	without	no	-	solitary	terminal or lateral
*Trentepohlia arborum*	no	without	no	basal	in cluster or solitary	sessile or lateral
*Trentepohlia annulata*	no	without	no	-	sessile	-
*Trentepohlia aurea*	no	without	no	basal	in cluster or solitary	sessile or lateral
*Trentepohlia iolithus*	no	without	no	-	in cluster or solitary	intercalary
*Trentepohlia umbrina*	no	without	no	distal	solitary	intercalary

#### *Phycopeltis aurea* Karsten, Ann. Jard. Bot. Buitenzorg 10: 1–66, 1891. (Figs. 1A–1C, 3A–3C, 4A–4D)

*Phycopeltis aurea* was commonly found on both sides of *Sansevieria trifasciata* Prain and *Dracaena angustifolia* Roxburgh leaves. The accession number is YN1220 (IHB).

The plants consist of discoidal thalli up to 1 mm in diameter. The brown-yellow appearance formed a distinct contrast to the green background. The circular alga is consisting of radiating, laterally appressed, dichotomous filaments (Figs. 1A, 1B, 4A and 4B). The sporangiate-laterals and gametangia are mainly intercalary in origin on sporophyte and gametophyte respectively (Figs. 1A, 4B and 4D). The thallus with both sporangia and gametangia was occasionally observed. Vegetative cells were 3.8–7.2×5.8–13.2 μm, length/width ratio is 1.0–2.2. Sporangia are 8.0–10.4×14.2–17.6 μm (Fig. 1C). The smaller length/width ratio of the regularly vegetative cells and the position of sporangia helped distinguish the species from *P. epiphyton*.

#### *Phycopeltis epiphyton* Millardet, Mem. Soc. Sci. Nat. Strasbourg 6:37–50, 1870. (Figs. 1D–1F, 3D–3F, 4E–4G)

The plants grew superficially on several substrata such as leaves (*Caryota ochlandra* Hance) (YN1201) and plastic tags (YN1228). Accession numbers are YN1201 (IHB) and YN1228 (IHB).

The algae are yellowish-green or orange discs formed by radiating and laterally appressed dichotomous filaments that usually have a crenate margin (occasionally a lobed margin). The individual plant can grow to 1.3 mm in diameter. The vegetative cells were 4.4–8.2 × 7.4–23.8 μm, mostly 5.5–8 × 10–21 μm, with a length/width ratio of 1.3 to 3.2 (mostly 1.8 to 2.8) (Figs. 1D, 4E and 4F). The sporangia-laterals of adult thalli are often produced from submarginal cells (Fig. 1E). The sporangia are 9.4–12.6 × 11.2–15.4 μm (Figs. 1F and 4G). The gametangia are produced from intercalary cells that are slightly raised over the surface of the thallus. Thalli of both gametangia and sporangia produced on the same plant are occasionally observed. Unfortunately, zoospores and gametes are not observed in our study.

The *P. epiphyton* specimens sampled from the botanical garden slightly differ from the original description and illustrations by Millardet (1870). Millardet stressed two features: the small size and the tendency to become reproductive at an early stage. However, many phycologists reported that individual *P. epiphyton* specimens range from 120–130 μm (Ettl & Gärtner 1995; Krishanmurthy 2000). The thallus can grow to 1.1 mm in diameter in the present study. Meanwhile, a previous study reported that British specimens of *P. epiphyton* grow up to 1 mm in diameter, which is rather consistent with our observation (John 2002). In our opinion, this phenomenon may be caused by the second or third growth (this phenomenon is also found in other *Phycopeltis* speices), thus the different size of thallus is comprehensible. We also found that reproductive structures can be formed at the early stage of the specimens used in our study. Other morphological characteristics and details of vegetative cells were in agreement with the description of Thompson and Wujek (1997).

#### *Phycopeltis flabellata* Thompson & Wujek, Trentepohliales: *Cephaleuros*, *Phycopeltis* and *Stomatochroon*. Morphology, taxonomy and ecology. Sci. Publ., Enfield, 81–2, 1997. (Figs. 1G, 2A–2C, 3G–3J, 4H, 5A–5B)

The alga was found only on the adaxial surface of *Mangifera indica* Linnaeus leaves. Accession number is GD1203 (IHB). A distinct contrast between the orange-gold appearance of the thalli and the bright-green leaves was easily observed.

Adult thalli consist of several to more than a dozen fan-like ramuli spreading or appressed to form more or less closed disks (sometimes perforate) (Figs. 1G and 5A) that can reach 2 mm in diameter, with a crenate margin. Ramuli are composed of dichotomous filaments (about 4–8 filaments) with one central-axis filament. The cells were 5–11.2 (average 8) × 11–30.6 μm (average 19) with a length/width ratio of 1.3 to 3.2 (Fig. 2A and 2B). The sporangiophores are formed by 2 cells. Sporangia are either reniform or sub-globular, 10–15 × 12–18 μm (Figs. 2C and 5B). The sporangia can be observed at the margin of the fanlike ramuli (Fig. 2C). Zoospores and gametes were not observed in present study.

*Phycopeltis flabellata* collected from the forest park approximately matched the morphological description of Thompson and Wujek (1997). However, our specimens have smaller vegetative cells (average 8 × 19 μm) compared with that of Rindi average 6.0–8.5 × 15–20 μm (Rindi *et al.* 2008a) and are very consistent with Thompson and Wujek’s description (average of 7.4 × 19 μm). In our observation, no significant difference in the shape and size of the three description of the vegetative cells were indicated. The problems of having closed thalli and no gametangia in Rindi’s specimens also occurred in our specimens. This similarity may suggest that both characteristics are caused by environmental factors and the vegetative cell size of this species slightly varies in different locations and environment.

#### *Phycopeltis prostrata* (De Wildem) Schmidle emend. Sarma, Nova Hedwigia Beih. 58:86–7, 1986. (Figs. 2E–2H, 3K–3O, 5C–5F)

Synonym: *Trentepohlia prostrata* De Wildem, Notarisia 11:84–91, 1896.

This species was observed growing on both sides of the leaves of several plants (mainly *Adiantum pedatum* Linnaeus) from the tropical rainforest. The accession number is YN1218 (IHB).

*Phycopeltis prostrata* were yellowish-green to pale-green and composed of branched filaments with disjunct margins that had erect hair. The prostrate vegetative filaments were loosely anastomose (Fig. 5C). The cells of the prostrate filaments were usually cylindrical and could be irregular, measuring 2.4–5 × 6–13.5 μm and a length/width ratio of 2.1–5.2 with about 3.6 on average. The erect filaments were easily observed on the gametophyte with usually four to seven cells and occasionally up to 10 cells (Figs. 2E and 5C). The erect filaments were abundant, unbranched and attenuated to a pointed or blunt apical cell, with a sessile gametangium on the basal cell (Figs. 2H, 5D). Sessile gametangia were spindly or ovoid, measuring 4.6–7.1 × 9.4–10.8 μm. Less erect hair were found on the sporophyte. Sporangiate-laterals (Figs. 2F, 2G, 5E, 5F) developed directly from intercalary cells. Sporangiophores were mainly composed of two cells and arose when growth restarted from the suffultory cells after an abundant discharge of zoosporangia. Sporangia were globular and measured 5.2–7.8 μm in diameter. Zoospores and gametes were not observed.

*Phycopeltis prostrata* was originally described by Wildeman in 1896 as *Trentepohlia prostrata* [39]. Schmidle (1897a) described a similar specimen with sporangia as a new species: *Hansgirgia polymorpha*. Schmidle (1897b) subsequently reported on the similarity between *H. polymorpha* and *T. prostrata*, and he thought they were same species, so he placed this entity in the genus *Phycopeltis* as *P. prostrata* [40]. Sarma (1986) amended the description of this species based on specimens collected from New Zealand. Some details of this alga were not reported in the characterization provided by Wildeman (1896) and Printz (1939) [41], such as the direct development of sporangia on few-celled stalks from prostrate filaments. However, most morphological characteristics in the present study match the description of Sarma (1986). The vegetative morphology of our material was in perfect agreement with the characterization by Sarma (1986). Therefore, the *P. prostrata* specimen was unambiguously determined.

### Developmental observations

*Phycopeltis flabellata*, *P. aurea* and *P. epiphyton* primarily grow by symmetric cell division, which was observed in our study (Fig. 3A–3O). The discoid thalli of the three species consisted of several (2–8) cells. *Phycopeltis aurea* symmetrically grew from one to hundreds of cells (Fig. 3A–3C). The primary development of *P. epiphyton* was also symmetrical (Fig. 3D–3F). More or less irregular discoids could be formed when plants were composed of about a hundred cells or fewer. The disc-like thalli were also very similar to *P. aurea*; however, the two species differed from each other in their size and length/width ratio. The fan-like development of *P. flabellata* occurred when the thalli were composed of > 20 cells (Fig. 3I). A noticeable fan-like ramullus could be found when > 36 cells were present (Fig. 3J). There was a slight difference among the three species at the early stage of spore or gamete germination. However, vegetative cells varied in shape when the thallus consisted of several to dozens of cells. The thallus of *P. prostrata* was not as symmetrical as the above mentioned three *Phycopeltis* species. The prostrate filaments of either young or adult thalli of *P. prostrata* anastomose loosely (Fig. 3K–3O). Erect hair were observed. The growth was not symmetrical during primary development, which contrasts with the other three species; another important difference between *Phycopeltis prostrata* and other three species was the dichotomous vegetative cells at primary development. It can be easily found that the vegetative cells were dichotomous when the thalli of *P. aurea*, *P. epiphyton*, and *P. flabellata* are consisted of only few cells, which was not found in *Phycopeltis prostrata*.

### Phylogenetic analyses (Figs. 6–7)

**Fig 6:**
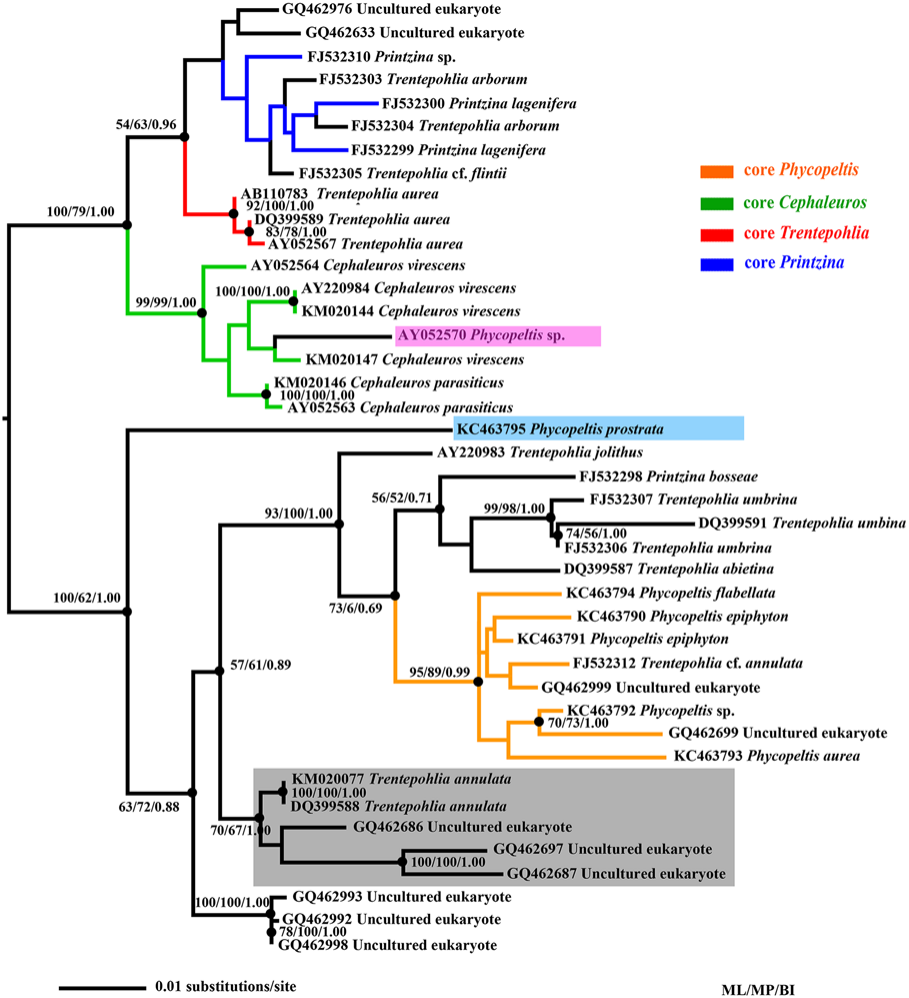
Phylogram inferred from ML analysis of SSU rDNA sequences in the Trentepohliales (obtained with RAxML, ln(L) = -4975.4). Numbers indicate branches highly supported under all inference methods (>50% BP, >0.5 PP). The caldes of core *Trentepohlia*, *Cephaleuros*, *Printzina* and *Phycopeltis* were colored in red, green, blue and orange respectively. The previous *Phycopeltis* sequence (Genbank no. AY052570) was shaded in pink, the *Phycopeltis prostrata* was shaded in cyan and *Trentepohlia annulata* group were shaded in grey.

**Fig 7:**
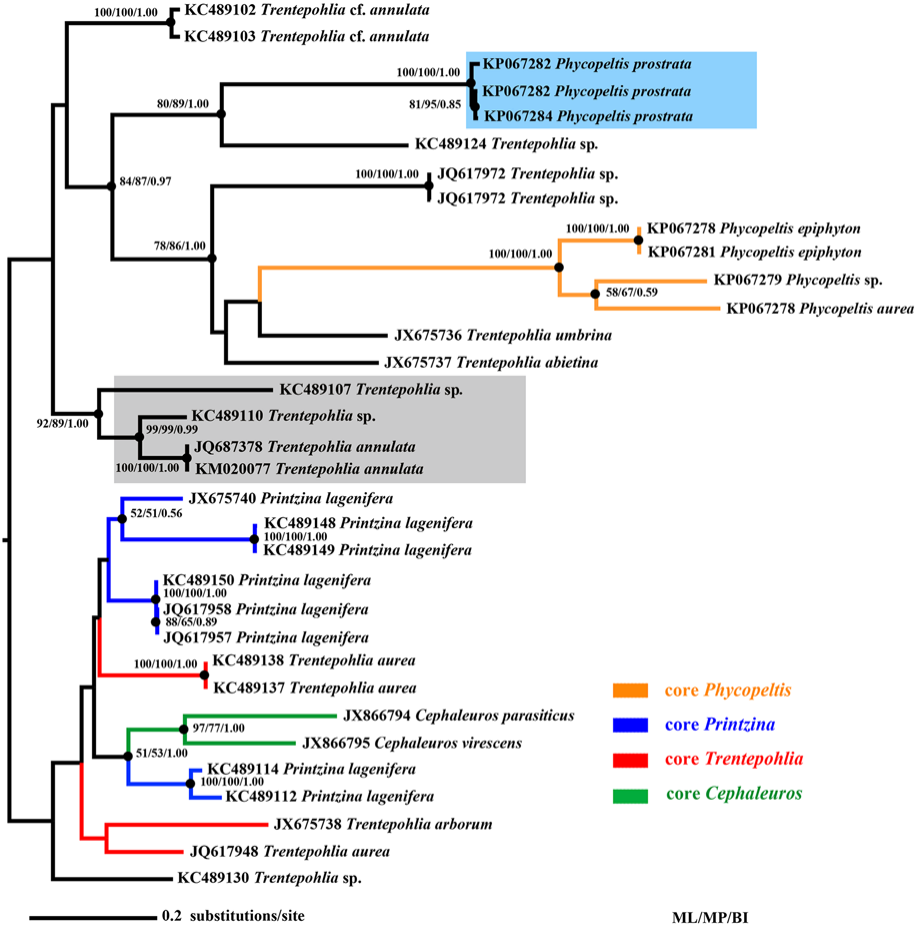
Phylogram inferred from ML analysis of ITS rDNA sequences in the Trentepohliales (obtained with RAxML, ln(L) = -8559.5). Numbers indicate branches highly supported under all inference methods (>50% BP, >0.5 PP). The clades of core *Trentepohlia*, *Cephaleuros*, *Printzina* and *Phycopeltis* were colored in red, green, blue and orange respectively. The *Phycopeltis prostrata* were shaded in cyan and *Trentepohlia annulata* group were shaded in grey.

We obtained six SSU rDNA sequecnes and seven ITS rDNA sequences of *Phycopeltis*. The nuclear SSU rDNA sequences aligned in this study were 1603 base pairs for the 41 taxon of Trentepohliaceae. A total of 201 sites (12.1%) among these nucleotides were variable, and 127 sites (7.9%) were parsimoniously informative. The base frequencies across taxa were homogeneous. The aligned ITS rDNA sequences were 789 base pairs, including 33 taxon of Trentepohliales. A total of 439 sites (55.6%) among these nucleotides were variable, and 371 sites (47.0%) were parsimoniously informative.

The two phylograms in present study showed similar topology with previous studies [42, 43, 44, 45]. The core groups (mainly consisted of their type species) of the four genera (*Trentepohlia*, *Cephaleuros*, *Printzina* and *Phycopeltis*) in Trentepohliales were easily found (Figs. 6 and 7) in three analyses of SSU and ITS rDNA. Obviously, the three genera *Printzina*, *Trentepohlia* and *Phycopeltis* were not monophyletic. The core *Phycopeltis* group (orange clade in both Figs. 6 and 7) showed a closest phylogenetic relationship with *T. umbrina* and *T. abietina* clade; the red clades (core *Trentepohlia* group) is monophyletic in SSU rDNA phylogram, but is polyphyletic in ITS rDNA phylogram. The blue clade (core *Printzina* group) is obviously polyphyletic in both phylograms. The strains of *Cephaleuros* (green clades in Figs. 6 and 7) in the phylogenetic analyses of this study represented a well-defined monophyletic group. *Trentepohlia annulata* and several environmental sequences clustered in one clade (grey-shaded in Figs. 6 and 7), on the basis of the main clade, which showed that there are lots of Trentepohliacean groups (may lichenized) still unknown. *T. annulata* is a special species since it is the single species that do not form sporangium-associated apparatus in its life history, which may be a good explain for this separated group.

## Discussion

### Taxonomic analyses

In previous research, many phycologists focused on one morphological feature, the position of the papilla-pore. According to this characteristic, Thompson and Wujek (1997) classified *T. umbrina* in *Phycopeltis* [2]. In fact, this papilla-pore do also occur in other species such as *Printzina bosseae*. In the present study, *P. prostrata* had a distant papilla-pore and morphology more like *Phycopeltis* than *T. umbrina*. However, if we examine other features, *P. prostrata* is more like *Trentepohlia.* For example, *P. prostrata* has a filament-like development rather than a disc-like thallus, it has a sessile gametangia on the basal cell of erect hair, and there was a greater cell length/width ratio. Conversely, most *Phycopeltis* species have a disc-like thallus and they have intercalary gametangia. Furthermore, when we examined the developmental morphology of young thalli, most *Phycopeltis* species had dichotomously branching filaments but *P. prostrata* lacked regularly dichotomously branched filaments. It can be easily found from Table 2 that the key characters of core *Phycopeltis* group distinguished from other group must include the dichotomously filaments either in early development or adult thalli. Some species such as *Trentepohlia umbrina* only with a distant papilla-pore should not be classified into *Phycopeltis*. Several species (such as *Phycopeltis irregularis*, *Phycopeltis minuta* and *Phycopeltis parva*) in *Phycopeltis* that do not show a dichotomously development may need a taxonomic revision.

### Phylogenetic analyses

Both *Cephaleuros* and *Phycopeltis* occur epiphytically on leaves. *Cephaleuros* is subcutically foliicolous by rhizoids; while *Phycopeltis* can also be found on non-living substrata without any anchoring cells. The two genera share several morphological characteristics such as fan-like ramuli and dichotomy of filaments but differ in key morphological features such as gametangium, sporangium, with/without rhizoids as well as shape and size of vegetative cells. These differences explain the early separation of *Phycopeltis* and the lack of close association with *Cephaleuros*, as determined by our phylogenetic analyses. Furthermore, the similar morphological characteristics may result from parallel evolution in the same or similar habitats. However, the previous published *Phycopeltis* sequence (Genbank no. AY052570, with a sequence length only 761 base-pairs, pink shaded in Fig. 6) falled within *Cephaleuros* group. The lack of genetic information caused by too short length or the contamination by other Trentepohliacean organism may explain this phenomenon.

The phylograms based on five SSU rDNA and seven ITS rDNA sequences obtained in our study clustered these *Phycopeltis* spp. (except *P. prostrata*) in one clade. This finding is attributed to features found only in *Phycopeltis.* These features include the equal dichotomy of filaments, monostromatic thalli composed of radiating and laterally appressed dichotomous filaments, distal escape pore of the sporangium and the solitary sporangiate-lateral on the suffultory cell. Most species from *Phycopeltis* are easily distinguished from other genera by these characteristics. *Phycopeltis prostrata* with a distant papilla-pore separated from other species such as *T. abietina*, *T. umbrina* and *T. arborum*, which indicates that this may be a characteristic of their ancestors, and do not only belong in the genus *Phycopeltis*. Both *Printzina bosseae* and *T. umbrina* also with a distant papilla-pore, however, they do not cluster within the core *Phycopeltis* group, and their thalli are not composed of dichotomous filaments, which once proved this characteristic is not generic feature of *Phycopeltis*. On the basis of the phylogeny and the unique morphology of *P. prostrata* and the core *Phycopeltis* group, the taxonomic position of *P. prostrata* need change, and should be classified into a new genus. Furthermore, phylogenetic analyses and morphological examination based on more or all species in this genus are needed. Thus, the traditional taxonomic criteria on genus *Phycopeltis* must be reassessed. And a new circumscription of the *Phycopeltis* with the erection of new genera could eventually be a useful solution to this paraphyly.

Important morphological characteristics used for delimitation at species-level such as fan-like or disc-like ramulli and the different positions of gametangia and sporangia are present in the four species. These characteristics occurring in these four species can represent most species in *Phycopeltis*. All phylogenetic analyses (ML, MP and BI) clustered these *Phycopeltis* species with branch values were 53, 90 and 0.99, respectively (Fig. 6). The four species and *Trentepohlia* cf. *annulata* clustered in the same clade indicated that this *Phycopeltis* (except *P. prostrata*) group may be monophyletic. We speculate that the specimen of *T*. cf. *annulata* may need a careful re-examination, or there may be a misidentification for this specimen. Furthermore, the SSU rDNA sequence was highly conserved, and this molecular marker may not be applicable to distinguish species in the genus. Thus, the phylogram based on ITS rDNA sequences (Fig. 7) once again proved that the core *Phycopeltis* group (except non-discoid thallus taxa such as *Phycopeltis prostrata*) was monophyletic.

The systematic position of some species without symmetrical thalli and ramulli, such as *P. irregularis*, *P. minuta* and *P. parva*, may not form a clade with *Phycopeltis* on the basis of developmental observation. Based on their loosely anastomosed and ravelling filamentous morphology, which is similar to *P. prostrata*, they may have similar developmental morphology to *P. prostrata*. The remaining species that possess equal-dichotomous filaments and coalescent disc-like thalli, which should be accepted as *Phycopeltis* (with type species *P. epiphyton*), may cluster with our four *Phycopeltis* spp.. New taxonomic solutions such as erection of a new genus may resolve this assumptive problem. However, this assumption requires further investigation based on more *Phycopeltis* species.

## Supporting Information

S1 Fig The abundant *Phycopeltis* on field plastic tags and leaves. S1A Fig. *Phycopeltis epiphyton* on plastic tags. S1B Fig. *Phycopeltis prostrata* on leaves of shade-requiring plants. S1C Fig. Showing the discoidal thalli formed by *Phycopeltis* sp. and fungi on leaves of *Caryota* sp.. S1D Fig. *Phycopeltis epiphyton* on leaves of *Caryota* sp.. Figs. S1E–F. *Phycopeltis aurea* on leaf blades of *Sansevieria* spp.. Fig. S1G. *Phycopeltis flabellata* on leaves of *Mitrephora* sp.
(TIF)

S1 File Alignments of SSU rDNA sequences used for phylogenetic analyses in present study. (FAS)

S2 File Alignments of ITS rDNA sequences used for phylogenetic analyses in present study. (FAS)

## References

[1] Neustupa J (2003) The genus *Phycopeltis* (Trentepohliales, Chlorophyta) from tropical Southeast Asia. Nova Hedwigia 76: 487–505. 10.1127/0029-5035/2003/0076-0487

[2] Thompson RH, Wujek DE (1997) Trentepohliales: Cephaleuros, Phycopeltis, and Stomatochroon. Morphology, Taxonomy and Ecology. Science Publishers, Enfield, New Hampshire 67 pp..

[3] Printz H (1939) Vorarbeiten zu einer Monographie der Trentepohliaceae. Nytt Magazin für Naturvidenskaberne 80:137–210.

[4] Good BH, Chapman RL (1978) Scanning electron microscope observations on zoosporangial abscission in *Phycopeltis epiphyton* (Chlorophyta). Journal of Phycology 14: 374–376. 10.1111/j.1529-8817.1978.tb00317.x

[5] Chapman RL, Good BH (1983) Subaerial symbiotic green algae: interactions with vascular plant hosts. In Goff L. J. (Ed.) Algal Symbiosis: A Continuum of Interaction Strategies. Cambridge University Press, New York 173 pp.

[6] Chapman RL (1984) An assessment of the current state of our knowledge of the Trentepohliaceae. In: Irvine D. E. G. and John D. M. (Eds), Systematics of the Green Algae. London and Orlando, Academic Press 233 pp.

[7] Rindi F, Guiry MD (2002a) The genus *Phycopeltis* (Trentepohliaceae, Chlorophyta) in Ireland: a taxonomic and distributional reassessment. Phycologia 41: 421–431. 10.2216/i0031-8884-41-4-421.1

[8] López-Bautista JM, Waters DA, Chapman RL (2002) The Trentepohliales revisited. Constancea 83 Available at: http://ucjeps.berkeley.edu/constancea/lopez_etal/trentepohliales.html (last accessed 19 February 2011).

[9] Neustupa J (2005) Investigation on the genus *Phycopeltis* (Trentepohliaceae, Chlorophyta) from South-East Asia, including the description of two new species. Cryptogamie Algologie 26: 229–232.

[10] Raven JA (1987) Biochemistry, biophysics and physiology of chlorophyll b-containing algae: implications for taxonomy and phylogeny. Progress in Phycology Research 5: 1–122.

[11] Sluiman H (1989) The green algal class Ulvophyceae: an ultrastructural survey and classification. Cryptogamic Botany 1: 83–94.

[12] Cocquyt E, Verbruggen H, Leliaert F, Clerck OD (2010) Evolution and cytological diversification of the green seaweeds (Ulvophyceae). Molecular Biology and Evolution 27: 2052–2061. 10.1093/molbev/msq091 20368268

[13] Nelsen MP, Plata ER, Andrew CJ, Lücking R, Lumbsch HT (2011) Phylogenetic diversity of Trentepohlialean algae associated with lichen-forming fungi. Journal of Phycology 47: 282–290. 10.1111/j.1529-8817.2011.00962.x 27021860

[14] Hametner C, Stocker-Wörgötter E, Rindi F, Grube M (2014) Phylogenetic position and morphology of lichenized Trentepohliales (Ulvophyceae, Chlorophyta) from selected species of Graphidaceae. Phycological Research 62: 170–186. 10.1111/pre.12055

[15] Hametner C, Stocker-Wörgötter E, Grube M (2014) New insights into diversity and selectivity of trentepohlialean lichen photobionts from the extratropics. Symbiosis 63:31–40. 10.1007/s13199-014-0285-z 25076805 PMC4110408

[16] Fritz L, Triemer RE (1985) A rapid simple technique utilizing Calcofluor WhiteM2Rfor the visualization of dinoflagellate thecal plates. Journal of Phycology 21: 662–664. 10.1111/j.0022-3646.1985.00662.x

[17] Liu G-X, Pei G-F, Hu Z-Y (2008) *Peridiniopsis niei* sp. nov. (Dinophyceae), a newspecies of freshwater red tide dinoflagellates from China. Nova Hedwigia 87: 487–499. 10.1127/0029-5035/2008/0087-0487

[18] Medlin L, Elwood HJ, Stickel S, Sogin ML (1988) The characterization of enzymatically amplified eukaryotic 16S-like rRNA-coding regions. Gene 71: 497–499. 10.1016/0378-1119(88)90066-2 3224833

[19] Vieira J, Messing J (1982) The pUC plasmids, an M13mp7-derived system for insertion mutagenesis and sequencing with synthetic universal primers. Gene 19: 259–268. 10.1016/0378-1119(82)90015-4 6295879

[20] Hayakawa Y, Ogawa T, Yoshikawa S, Ohki K, Kamiya M (2012) Genetic and ecophysiological diversity of Cladophora (Cladophorales, Ulvophyceae) in various salinity regimes. Phycological Research 60: 86–97. 10.1111/j.1440-1835.2012.00641.x

[21] Thompson JD, Gibson TJ, Plewniak F, Jeanmougin F, Higgins DG (1997) The ClustalX windows interface: flexible strategies for multiple sequence alignment aided by quality analyses tools. Nucleic Acids Research 25: 4876–4882. 10.1093/nar/25.24.4876 9396791 PMC147148

[22] Gouy M, Guindon S, Gascuel O (2010) SeaView version 4: a multiplatform graphical user interface for sequence alignment and phylogenetic tree building. Molecular Biology and Evolution 27: 221–224. 10.1093/molbev/msp259 19854763

[23] Tamura K, Peterson D, Peterson N, Stecher G, Nei M, et al (2011) MEGA5: Molecular Evolutionary Genetics Analyses Using Maximum Likelihood, Evolutionary Distance, and Maximum Parsimony Methods. Molecular Biology and Evolution 28: 2731–2739. 10.1093/molbev/msr121 21546353 PMC3203626

[24] Swofford DL (2002) PAUP*: Phylogenetic analyses using parsimony (and other methods), version 4.0 Beta. Sunderland, Massachusetts: Sinauer.

[25] Stamatakis A (2014) RAxMLversion 8: a tool for phylogenetic analysis and post-analysis of large phylogenies. Bioinformatics 30: 1312–1313. 10.1093/bioinformatics/btu033 24451623 PMC3998144

[26] Huelsenbeck JP, Ronquist F (2001) MRBAYES: Bayesian inference of phylogenetic trees. Bioinformatics 17: 754–755. 10.1093/bioinformatics/17.8.754 11524383

[27] Posada D, Crandall KA (1998) Modeltest: Testing the model of DNA substitution. Bioinformatics. 14: 817–818. 10.1093/bioinformatics/14.9.817 9918953

[28] Allali HA, Rindi F, López-bautista JM (2013) Biodiversity of Trentepohliales (Ulvophyceae, Chlorophyta) in Gabon, Central Africa. Nova Hedwigia 96: 309–324. 10.1127/0029-5035/2013/0096

[29] Rindi F, Guiry MD (2002b) Diversity, life history, and ecology of *Trentepohlia* and *Printzina* (Trentepohliales, Chlorophyta) in urban habitats in western Ireland. Journal of Phycology 38: 39–54. 10.1046/j.1529-8817.2002.01193.x

[30] Rindi F, Guiry MD, Critchley AT, Gall EAR (2003) The distribution of some species of Trentepohliaceae (Trentepohliales, Chlorophyta) in France. Cryptogamie Algologie 24: 133–144.

[31] Rindi F, Menéndez JL, Guiry MD, Rico JM (2004) The taxonomy and distribution of *Phycopeltis* (Trentepohliaceae, Chlorophyta) in Europe. Cryptogamie Algologie 25: 3–17.

[32] Rindi F, Sherwood AR, Guiry M (2005) Taxonomy and distribution of *Trentepohlia* and *Printzina* (Trentepohliales, Chlorophyta) in the Hawaiian Islands. Phycologia 44: 270–284. 10.2216/0031-8884(2005)44[270:TADOTA]2.0.CO;2

[33] Rindi F, Guiry MD, López-bautista JM (2006) New records of Trentepohliales (Ulvophyceae, Chlorophyta) from Africa. Nova Hedwigia 83: 431–449. 10.1127/0029-5035/2006/0083-0431

[34] Rindi F, López-bautista JM (2008a) Diversity and ecology of Trentepohliales (Ulvophyceae, Chlorophyta) in French Guiana. Crytogamie Algologie 29: 13–43.

[35] Rindi F, Lam DW, López-bautista JM (2008b) Trentepohliales (Ulvophyceae, Chlorophyta) from Panama. Nova Hedwigia 87: 421–444. 10.1127/0029-5035/2008/0087-0421

[36] Sarma P (1986) The freshwater Chaetophorales of New Zealand. Nova Hedwigia 58: 1–169.

[37] Thompson RH, Wujek DE (1992) Printzina gen.nov. (Trentepohliaceae), including a description of a new species. Journal of Phycology 28: 232–237. 10.1111/j.0022-3646.1992.00232.x

[38] Wildeman DÉ (1896) Les espèces du genre *Trentepohlia* . Notrisia 11: 84–91.

[39] López-bautista JM, Waters DA, Chapman RL (2003) Phragmoplatin, green algae and the evolution of cytokinesis. International Journal of Systematic and Evolutionary Microbiology 53: 1715–1718. 10.1099/ijs.0.02561-0 14657098

[40] Schmidle W (1897a) Einige Baumalgen aus Samoa. Hedwigia 36: 277–287.

[41] Schmidle W (1897b) Vier neue von Professor Lagerheim in Ecuador gesammelte Baumalgen. Berichte der Deutschen Botanischen Gesellschaft 15: 456–459.

[42] Boedeker C, Karsten U, Leliaert F, Zuccarello GC (2013) Molecular, biochemical and morphological data suggest an affiliation of Spongiochrysis hawaiiensis with the Trentepohliales (Ulvophyceae, Chlorophyta). Phycological research 61: 133–144. 10.1111/pre.12011

[43] López-bautista JM, Rindi F, Guiry MD (2006) Molecular systematics of the subaerial green algal order Trentepohliales: an assessment based on morphological and molecular data. International Journal of Systematic and Evolutionary Microbiology 56: 1709–1715. 10.1099/ijs.0.63990-0 16825655

[44] Rindi F, Lam DW, López-bautista JM (2009) Phylogenetic relationship and species circumcription in *Trentepohlia* and *Printzina* (Trentepohliales, Chlorophyta). Molecular and Phylogenetics Evolution 52: 329–339. 10.1016/j.ympev.2009.01.009 19489121

[45] Suutari M, Majaneva M, Fewer DP, Voirin B, Aiello A, et al (2010) Molecular evidence for a diverse green algal community growing in the hair of sloths and a specific association with *Trichophilus welckeri* (Chlorophyta, Ulvophyceae). BMC Evolutionary Biology 10: 86–97. 10.1186/1471-2148-10-86 20353556 PMC2858742

