# Supplementary figures and images for: Morphological Examination and Phylogenetic Analyses of Phycopeltis spp. (Trentepohliales, Ulvophyceae) from Tropical China
Source: PLoS One. 2015 Feb 2;10(2):e0114936. doi: 10.1371/journal.pone.0114936 (PMC4314078; doi:10.1371/journal.pone.0114936)

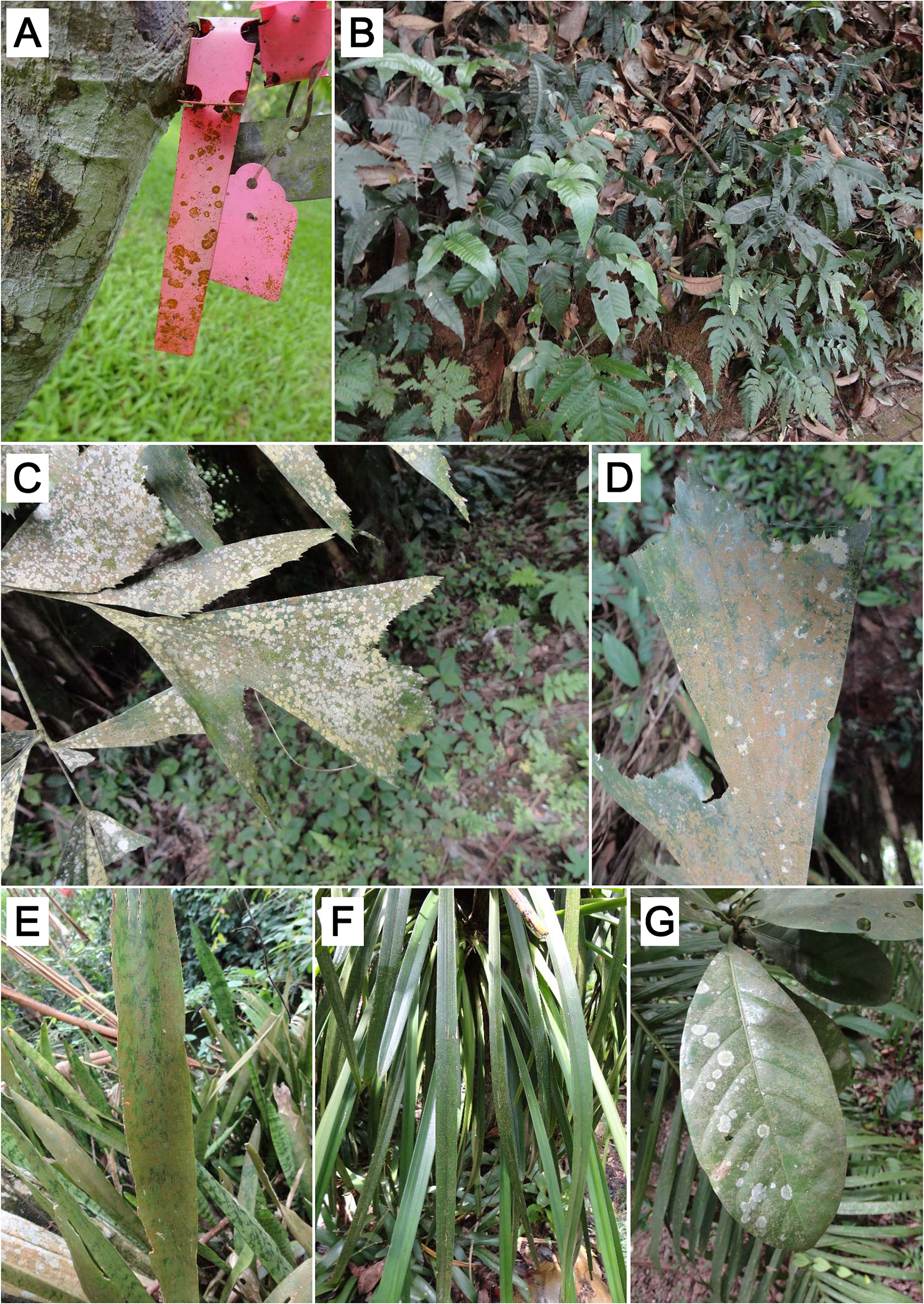

Supplement: S1 Fig — Phycopeltis epiphyton on plastic tags. S1B Fig. Phycopeltis prostrata on leaves of shade-requiring plants. S1C Fig. Showing the discoidal thalli formed by Phycopeltis sp. and fungi on leaves of Caryota sp.. S1D Fig. Phycopeltis epiphyton on leaves of Caryota sp.. Figs. S1E–F. Phycopeltis aurea on leaf blades of Sansevieria spp.. Fig. S1G. Phycopeltis flabellata on leaves of Mitrephora sp. (TIF) [file pone.0114936.s001.tif]
